# A retrospective database study on 2-year weight trajectories in first-episode psychosis

**DOI:** 10.3389/fpsyt.2023.1185874

**Published:** 2023-06-27

**Authors:** Yi Chian Chua, Edimansyah Abdin, Charmaine Tang

**Affiliations:** ^1^Department of Psychosis, Institute of Mental Health, Singapore, Singapore; ^2^Research Division, Institute of Mental Health, Singapore, Singapore

**Keywords:** weight gain, first-episode psychosis, intervention, trajectories, outcomes

## Abstract

**Introduction:**

It is critical to focus on individual weight profiles in line with efforts to tailor treatment, given the heterogeneous nature of the clinical population. This study aims to identify and describe possible two-year weight trajectories among patients accepted to the Early Psychosis Intervention Programme (EPIP) in Singapore.

**Methods:**

De-identified data was extracted from EPIP’s standing database for patients accepted from 2014 to 2018 with a schizophrenia spectrum disorder. Data collected at fixed time-points (baseline, 1-year, and 2-year) included anthropometric measures (height and weight), and sociodemographic (age, sex, highest education level, and vocational status) and clinical (duration of untreated psychosis, number of inpatient admissions, and scores on the Positive and Negative Syndrome Scale and Global Assessment of Functioning) information.

**Results:**

A total of 391 complete data sets were included for main analyses. Those with missing weight data were more likely to be males, older at baseline, have a highest education level of tertiary and above at baseline, and have a longer duration of untreated psychosis. The weight change across two years resulted in the following membership breakdown: 151 (38.6%) in super high risk; 133 (34.0%) in high risk mitigated; 17 (4.3%) in at risk; 34 (8.8%) in delayed risk; and 56 (14.4%) in low risk.

**Discussion:**

The lack of pharmacological, dietary, and physical activity data is a significant limitation in this study; however, the results reinforce the justification for future studies to prospectively capture and examine the influence of these data, with the aim of early detection and weight intervention for high risk groups.

## 1. Introduction

It has been well-documented that people with psychosis are at risk of developing metabolic syndrome and cardiovascular diseases during the course of antipsychotic treatment. This can largely be explained by antipsychotic-induced weight gain, which has been associated with resulting higher body mass index (BMI), and increased rates of morbidity and mortality (1, 2). A prospective study of people with first-episode psychosis (3) identified that majority of the participants with schizophrenia (62%) and bipolar (50%) ended up obese 20 years after their first hospitalization for psychosis, with related physical health complications. A meta-analysis by Bak and colleagues (4) had shown robust findings that antipsychotic use was linked to significant weight gain in antipsychotic-naïve patients, and that this weight gain was independent of psychiatric diagnoses. In the local Singaporean context, antipsychotic-induced weight gain was observed to take place in the majority (79.2%) of a young adult population, in a short span of 6 months from baseline (5). It was also found that upon matching healthy controls to patients receiving treatment at the Early Psychosis Intervention Programme (EPIP) in Singapore, the patients had a higher prevalence of diabetes even though they had lower BMI than controls at baseline (6). This, together with the low prevalence of obesity among the patients at illness onset, seems to suggest that the abnormal glucose metabolism was likely an associated result of the antipsychotic treatment.

However, while there has been literature examining antipsychotic-induced weight gain in drug-naïve patients, it is also critical to focus on individual weight profiles over time in line with efforts to tailor intervention. The heterogeneous nature of this clinical population has already been well-studied in terms of illness features; for example, a local study by Abdin and colleagues (7) had elicited among their sample of patients with first-episode psychosis, discrete trajectories of psychopathology and functioning over 2-years of follow-up, with certain trajectories associated with higher risk of deterioration as compared to the rest. Internationally, symptom trajectories over longer periods of time have also been identified (8, 9). Meanwhile, Zheng and colleagues (10) had derived latent BMI trajectories in a large non-clinical sample of retired older adults, which were found to be more effective at predicting mortality risk than static BMI status. It stands to reason that investigating distinct individual weight gain patterns in clinical populations would also be beneficial and yield significant information in preventing adverse physical health events, and maximizing treatment utility for these patients. Previous research suggest that the first year of antipsychotic treatment is often the most critical, as it is where majority of the weight gain happens and is sustained over the next few years (11, 12). These early weight changes were associated with persistent adverse metabolic effects later on, such as high triglyceride levels, and were able to predict further weight gain (13). An important qualitative study by Waite and colleagues (14) highlighted that weight gain profoundly impacted participants’ sense of self-worth, and left them with a loss of control, hope, and motivation for interventions. Given their youth and the fact that antipsychotic treatment would potentially be required for a long period of time, the participants also shared their preference for early conversations on common weight gain trajectories to expect.

Weight interventions often have to be labor-and resource-intensive in order to be truly effective, which may result in costs too high to be implemented indiscriminately (15, 16). Therefore, such interventions may only be considered feasible when applied to patients identified to be in severe need of such weight management services. Even so, the type of intervention provided should be tailored accordingly. For those at-risk for severe weight gain, preventative measures such as psychoeducation and nutrition counselling should be supplied. Meanwhile, those already with severe weight gain should be titrated onto a lower-risk antipsychotic or prescribed with supplementary medication to mitigate weight gain, and at the same time, provided with more intensive support such as lifestyle intervention and specialist physical healthcare (17). As such, international guidelines have been published to improve routine monitoring of cardiometabolic parameters in patients newly initiated to antipsychotic treatment (18, 19). Timely monitoring of potential adverse metabolic events will aid clinicians in making optimal decisions to mitigate antipsychotic treatment side effects. Despite these concerns, challenges still impede efforts to conduct physical health monitoring regularly, and discrepancies still remain between recommended standards and clinical practice (20).

The primary aims of the current study are thus to: (a) identify and describe possible 2-year weight trajectory patterns among patients accepted to EPIP in Singapore; and (b) highlight sociodemographic differences, if any, between those with complete 2-years of weight data and those without. Implications and recommendations for future directions will also be discussed.

## 2. Materials and methods

EPIP is an intensive intervention program in Singapore for first-episode psychosis, led by a multidisciplinary team including psychiatrists and allied health professionals. Patients accepted into the program fulfil the following criteria: (a) age between 16 and 40 years inclusive, (b) first-episode psychotic disorder with no prior or minimal treatment, and (c) psychotic disorder that is not secondary to a general medication condition or substance use. As part of EPIP’s service evaluation efforts, patient sociodemographic and clinical data are routinely collected at fixed time points (baseline, 1-year, and 2-year) and entered into a standing database (DSRB Reg. No.: IMH-2004-001) registered with the National Healthcare Group (NHG) Domain Specific Review Board (DSRB). The ratings used to collect each patient’s data are completed by the case managers in charge of their care, as well as their treating clinicians. Patients receiving treatment attend follow-up sessions at the outpatient clinic at the Institute of Mental Health (IMH), where their anthropometric measures – such as height and weight, and the resulting calculated BMI – are usually taken prior to their consultation session. These anthropometric measures are also taken when they are warded as an inpatient, and are cross-captured in the standing database through the yearly sociodemographic assessment by the case managers.

For this retrospective database study, ethics approval was also obtained from NHG DSRB (Ref. No.: 2020/01388). De-identified data was extracted from the EPIP standing database for all patients accepted into the program between 01 Jan 2014 and 31 Dec 2018 inclusive. Patients were not re-contacted and no data was extracted from individual medical records. Sociodemographic variables of interest included age at baseline, gender, and highest education level at baseline, and vocational status at baseline and 2-year. Highest education level was regrouped into two levels: tertiary and above vs. below tertiary; similarly, vocational status was regrouped into two categories: meaningfully occupied in an age-appropriate role (e.g., gainfully employed, homemaker, students, etc.) vs. unemployed. Clinical variables collected included duration of untreated psychosis (DUP), number of inpatient admissions, Positive and Negative Syndrome Scale (PANSS) scores at baseline and 2-year, and Global Assessment of Functioning (GAF) disability scores at baseline and 2-year. Number of inpatient admissions was regrouped into three levels: no admissions vs. one admission vs. multiple admissions. Lastly, anthropometric measures extracted included height at baseline, and weight at baseline, 1-year, and 2-year. To ensure a homogenous sample for data analysis and compensate for the lack of medication data, only patients with a diagnosis of a schizophrenia spectrum disorder were included for analysis. Based on clinical experience, these patients would most likely be prescribed with only antipsychotics, as compared to patients with a diagnosis of affective psychosis, where prescriptions would likely include anti-depressants or mood stabilizers. They were also likely to be on longer term antipsychotics as compared to patients with brief psychotic disorder, who may be on a shorter course or lower dosage of antipsychotics.

**Figure 1:**
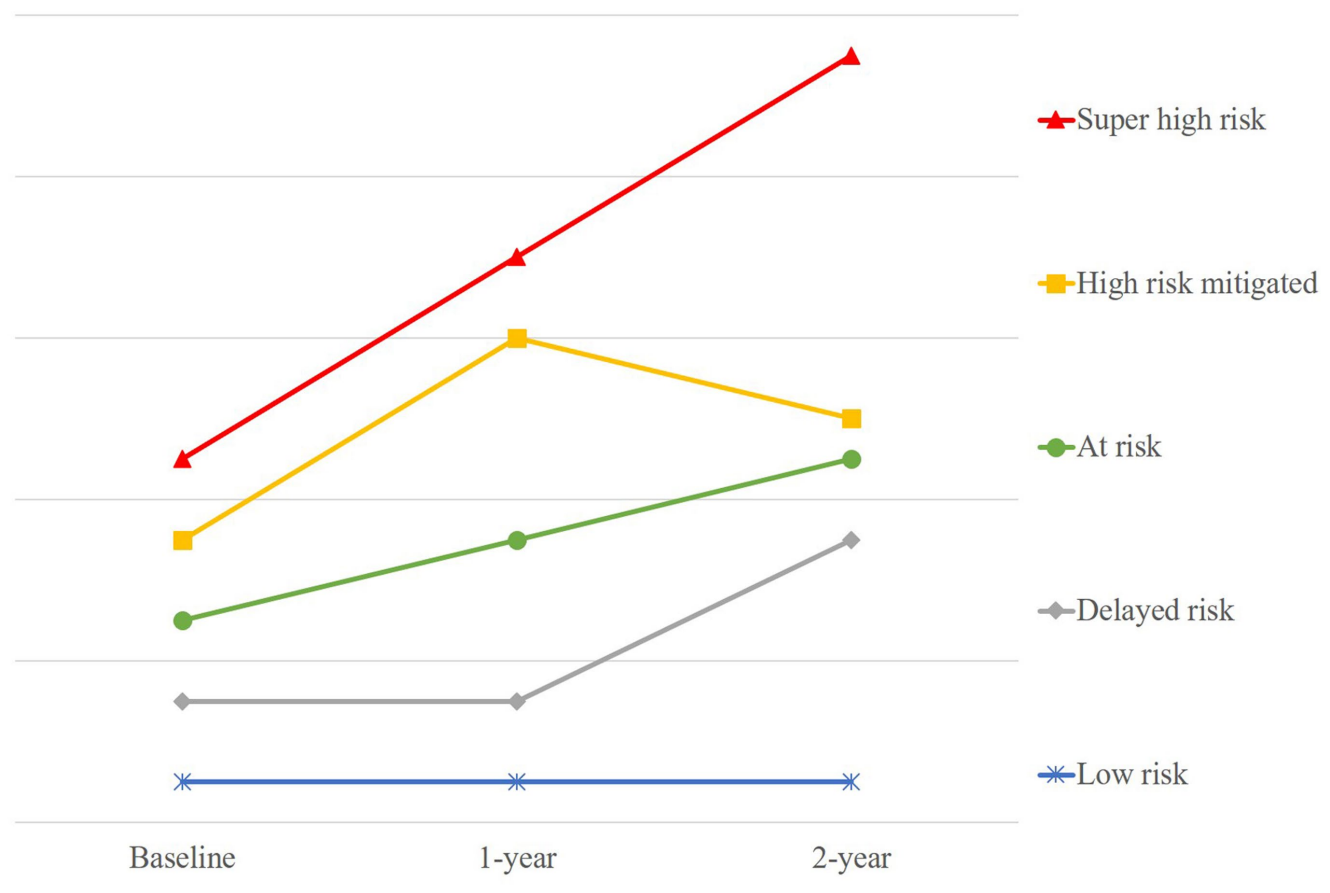
Simplified visual representation of the five different weight gain trajectories over 2-years.

Statistical analyses were conducted using IBM SPSS 23. Mean and standard deviations were computed for continuous variables, and frequencies and percentages were computed for categorical variables. To fulfil the study’s main aims, baseline sociodemographic characteristics between those who did and did not have complete weight data for the first 2-years were compared using *t-* and chi-square tests. Subsequently, data sets with complete clinical and weight data were used for primary analyses. Clinically significant weight gain was defined as ≥7% for this study (21). Percentage weight changes between each time point were thus grouped into four categories: (a) increase severe, for weight gain ≥7%; (b) increase mild, for weight gain <7 and > 1%; (c) maintain, for weight gain ≤1% and weight loss ≤1%; and (d) decrease, for weight loss >1%. Following, each included data set was assigned a weight profile according to their weight change categories across the first 2-years: (a) super high risk (e.g., increase severe across 2-years); (b) high risk mitigated (e.g., increase severe then decrease); (c) at risk (e.g., increase mild across 2-years); (d) delayed risk (e.g., maintain then increase severe); and (e) low risk (e.g., maintain across 2-years). The exact grouping matrix is presented in Table 1, while a simplified visual representation of each weight trajectory is depicted in Figure 1. Sociodemographic and clinical variables between the different groups of weight gain by the first year, and between those who continued to gain significant weight through to the second year and those who did not, were also compared using *t*-tests and one-way ANOVAs for continuous variables and chi-square tests for categorical variables, to explore if there were any other potential protective or risk factors to differentiate between these groups.

**Table 1:** Grouping matrix and membership of the different weight trajectory profiles.

BL → 1-yr	1-yr → 2-yr				
	Increase severe (gain ≥7%)	Increase mild (1% < gain <7%)	Maintain (gain ≤1% & loss ≤1%)	Decrease (loss >1%)	Total (row)
Increase severe (gain ≥7%)	Super high risk 57 (14.6%)	Super high risk 79 (20.2%)	High risk mitigated 34 (8.7%)	High risk mitigated 99 (25.3%)	269 (68.8%)
Increase mild (1% < gain <7%)	Super high risk 15 (3.8%)	At risk 17 (4.3%)	Low risk 9 (2.3%)	Low risk 29 (7.4%)	70 (17.9%)
Maintain (gain ≤1% & loss ≤1%)	Delayed risk 5 (1.3%)	Delayed risk 3 (0.8%)	Low risk 3 (0.8%)	Low risk 3 (0.8%)	14 (3.6%)
Decrease (loss >1%)	Delayed risk 16 (4.1%)	Delayed risk 10 (2.6%)	Low risk 3 (0.8%)	Low risk 9 (2.3%)	38 (9.7%)
Total (column)	93 (23.8%)	109 (27.9%)	49 (12.5%)	140 (35.8%)	391 (100%)

BL, Baseline; 1-yr: 1-year; 2-yr: 2-year.

Separately, as part of secondary exploratory analyses, 2-year clinical (PANSS and GAF scores) and vocational (meaningfully occupied vs. unemployed) outcomes were compared between each separate weight gain trajectory using chi-square test or one-way ANOVAs, and linear regression and binary logistic analyses were used to evaluate the contribution of the weight trajectory classification on these outcomes, after accounting for other sociodemographic and clinical factors. This was conducted in the hopes of preliminarily validating differences in 2-year outcomes amongst the identified separate weight trajectories. Statistical significance was established at *p* < 0.05.

## 3. Results

A total of 686 patients were accepted into EPIP from 01 Jan 2014 to 31 Dec 2018 with a diagnosis of a schizophrenia spectrum disorder and completed at least 2-years of the program. However, only 445 (64.9%) had complete weight data for the first 2-years. Those with missing data were more likely to be males, *χ*^2^ (1, *N* = 686) = 7.3, *p* = 0.007; older at baseline, *t* (684) = 3.491, *p* = 0.001; have a baseline highest education level of tertiary and above, *χ*^2^ (1, *N* = 678) = 15.2, *p* < 0.001; and have a longer DUP, *t* (453) = 2.525, *p* = 0.012. There were no significant differences between those who had complete weight data for the first 2-years and those without, in terms of baseline vocational status, PANSS total and GAF disability scores, and number of inpatient admissions. The mean (SD) weight gain over the 2-years was 9.7 (9.6) kg, with 42 (9.4%) participants gaining 40% and above of their baseline weight, and 124 (27.9%) gaining 20–40% of their baseline weight. Using the Asia-Pacific modified BMI classification by the World Health Organization (22), at the end of the 2-years, 210 (47.2%) were obese (BMI ≥25 kg/m^2^), 65 (14.6%) were overweight (23 kg/m^2^ ≤ BMI <25 kg/m^2^), 133 (29.9%) were normal (18.5 kg/m^2^ ≤ BMI <23 kg/m^2^), and 37 (8.3%) were underweight (BMI <18.5 kg/m^2^).

**Figure 2:**
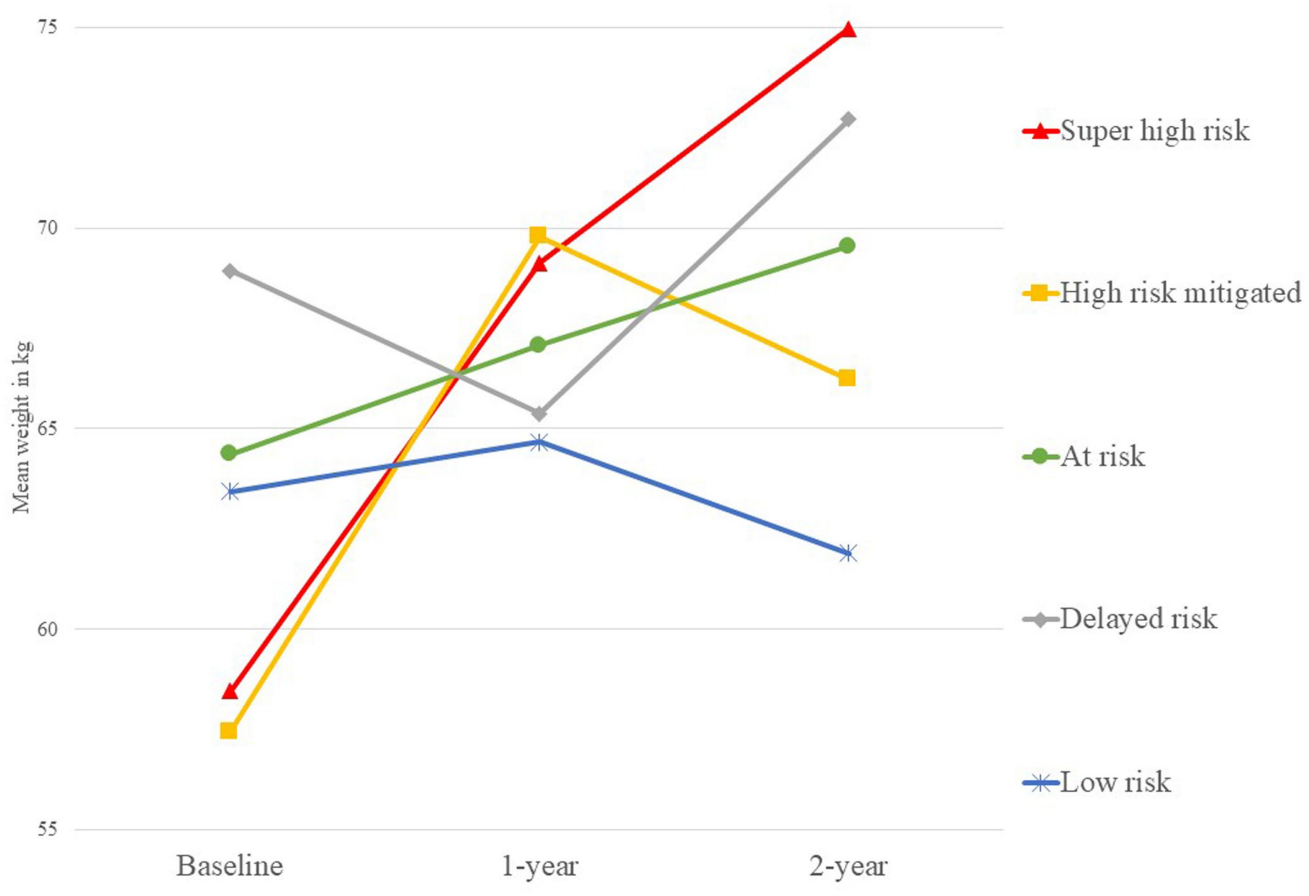
Actual mean weight of the five different weight gain trajectories over 2-years.

Of the 445 data sets with complete weight data for the first 2-years, 391 (87.9%) had complete clinical data and were included for the main analyses. Sociodemographic and clinical information of these are presented in Table 2. The recorded weight change across 2-years resulted in the following weight profile membership: 151 (38.6%) total in super high risk; 133 (34.0%) total in high risk mitigated; 17 (4.3%) total in at risk; 34 (8.8%) total in delayed risk; and 56 (14.4%) total in low risk. A detailed breakdown of the group membership had also been included in Table 1, and the actual mean weight of each group presented in Figure 2. Meanwhile, one-way ANOVAs and chi-square tests between the different groups of weight gain by the first year showed that there were no significant differences between these two groups, except for number of inpatient admissions, *χ*^2^ (6, *N* = 391) = 23.6, *p* = 0.001. There were also no statistically significant differences found between those who continued to gain weight from the first to second year and those who did not, amongst those who had significant weight gain from baseline to the first year.

**Table 2:** Sociodemographic and clinical information of datasets included for main analyses.

	Total (*n* = 391)
Age at baseline – years, mean (SD)	25.8 (6.7)
Gender – no. (%)	
Male	193 (49.4)
Female	198 (50.6)
Highest education level at baseline – no. (%)	
Tertiary and above	204 (52.2)
Below tertiary	187 (47.8)
Vocational status at baseline – no. (%)	
Meaningfully occupied in an age-appropriate role	202 (51.7)
Unemployed	189 (48.3)
DUP – months, mean (SD)	14.4 (22.3)
No. of inpatient admissions in total – no. (%)	
No admissions	91 (23.3)
One admission	130 (33.2)
Multiple admissions	170 (43.5)
PANSS scores at baseline – mean (SD)	
Total	84.1 (22.5)
Positive	22.8 (6)
Negative	19 (8.6)
General psychopathology	42.4 (12.4)
GAF disability score at baseline – mean (SD)	42.6 (12.2)
Weight at baseline – kg, mean (SD)	60.0 (16.0)
BMI at baseline – kg/m^2^, mean (SD)	22.1 (5.2)
Vocational status at 2-year – no. (%)	
Meaningfully occupied in an age-appropriate role	243 (62.1)
Unemployed	148 (37.9)
PANSS scores at 2-year – mean (SD)	
Total	42.7 (13.5)
Positive	9.2 (3.4)
Negative	11.3 (5.9)
General psychopathology	22.2 (6.6)
GAF disability score at 2-year – mean (SD)	71.4 (10.1)
Weight at 2-year – kg, mean (SD)	69.7 (19.0)
BMI at 2-year – kg/m^2^, mean (SD)	25.5 (6.1)

DUP, duration of untreated psychosis; PANSS, Positive and Negative Syndrome scale; GAF, Global Assessment of Functioning; BMI, body mass index.

In addition, secondary exploratory analyses were conducted and the results are as follows. It was found that the delayed risk weight trajectory was consistently scoring higher on the 2-year PANSS positive and general psychopathology subscales and consistently scoring lower on the 2-year GAF disability scores as compared to the other groups (Supplementary Table S1). However, there was no significant difference in 2-year vocational status (meaningfully engaged in an age-appropriate role vs. unemployed) between the weight trajectory groups. Follow-up linear regressions revealed that belonging to the delayed risk weight trajectory group (vs. belonging to the super high risk, high risk mitigated, or at risk weight trajectory groups) and having multiple inpatient admissions significantly predicted 2-year PANSS total score (Supplementary Table S2) and lower GAF disability scores (Supplementary Table S3), after accounting for other variables (age, gender, highest education level, DUP, and baseline PANSS total and GAF disability scores).

## 4. Discussion

In a 2-year period of follow-up, majority of the study sample, made up of drug-naïve patients with a diagnosis of first-episode schizophrenia spectrum disorder, gained a significant amount of weight, which was congruent with pre-existing literature on the topic (5, 11, 23, 24). One of the primary aims of the present study was to identify and describe discrete 2-year weight trajectory patterns among patients accepted into EPIP in Singapore. It was revealed that majority of the patients with complete weight and clinical data belonged to the super high risk (38.6%) and high risk mitigated (34.0%) groups. The main difference between these two groups was that while both trajectories demonstrated clinically significant weight gain (≥7%) in the first year, the high risk mitigated group managed to maintain or lose weight while the super high risk group continued to gain weight during the second year. Therefore, it was of interest to examine if there were potential sociodemographic differences between these two groups, in hopes of elucidating possible protective factors. There was a lack of significant differences found between these two groups; however, their differences may possibly lie beyond variables collected in this study. Zeroing in on weight gain patterns during the first year, those in the increase severe group (vs. increase mild, maintain, or decrease groups) were more likely to have one or multiple inpatient admissions instead of none. It is possible that patients with more severe psychopathology required more episodes of inpatient treatment, and the resultant increased use of psychotropic medications may have accounted for the significant weight gain. Conversely, significant weight gain may also be a surrogate marker heralding a more sinister course of illness in that patients with more severe psychopathology and who eventually have more inpatient episodes, may have had poorer self-care, dietary habits, and more sedentary lifestyles to begin with. Interestingly, we also found that the delayed risk group, out of the five trajectory groups, had the worst symptomatology and functioning at the end of the 2-years. We postulate that the delayed risk group may have had symptoms inadequately addressed in the first year of treatment, resulting in significant weight gain in the second year of treatment, and ultimately poorer clinical outcomes at the end of 2-years.

In the present study, there are hospital-wide processes in place for the collection of anthropometric data for patients in both the in-and out-patient settings. However, despite best efforts, these monitoring protocols are often not adhered to at the outpatient clinics for a variety of systemic and individual patient-/clinician-related reasons. This issue is not unique to our setting. A retrospective national audit in the United Kingdom of records of patients with schizophrenia or schizoaffective disorder revealed that BMI documentation was lacking even for those with established cardiovascular disease history (25). A recent commentary by Azfr Ali and colleagues (26) discussed some potential practical challenges in balancing cardiometabolic monitoring with antipsychotic treatment. Therapeutic response and risk of relapse are often primary drivers of the decision over which antipsychotic to use in treatment, which may result in overlooking of the antipsychotic’s associated adverse profiles. Furthermore, treatment resistance is a major concern during early stages of psychosis treatment (27, 28), making addressing poor illness insight and adherence to treatment a priority over fulfilling the standards for cardiometabolic monitoring. The implications of the weight data for the first 2-years not missing at random include the possibility that analyses in this paper may have been biased, and the secondary exploratory results should be interpreted with caution. Despite these real-world limitations inherent in our naturalistic study, the weight data not missing at random provided some valuable insights into existing gaps in weight monitoring procedures and allowed us to better understand the types of patients who were less likely to have their anthropometric measurements monitored. Firstly, males were more likely to have missing data, which may be due to having less concerns about weight or image. However, this has important repercussions as males tend to engage in poorer lifestyle behavior (29) and develop cardiovascular disease at a younger age (30). Secondly, those older and having a highest education level of tertiary and above at baseline may be functioning better and less likely to attend outpatient appointments where the weight monitoring would occur. Conversely, those with a longer DUP may also be at high risk of defaulting outpatient appointments because of poorer illness insight or more severe symptoms. Work should be put into evaluating points of contact with the patient, and how to increase chances of successfully collecting data on their anthropometric measures (31, 32).

The lack of pharmacological, dietary, and physical activity data is a significant limitation in this study. Pillinger and colleagues (33) compared the effects of 18 antipsychotics on metabolic function, with olanzapine and clozapine ranked the worst, while others like aripiprazole ranked the most benign. Hence, the type of antipsychotic and dosage prescribed or compliance to medication could have had an impact on the resulting weight gain pattern experienced by an individual (34). Meanwhile, a scoping review described preliminary findings from food surveys and self-report questionnaires that antipsychotic exposure was associated with increased disinhibition in terms of appetite and snacking, leading to disturbed eating behaviors over longer periods of time (35). Another meta-analysis also found that people with severe mental illness were significantly more sedentary and spent less time doing moderate or vigorous physical activity than matched healthy controls (36). Therefore, in an attempt to control the undue influence of unmeasured variables, a homogeneous sample of patients of patients with a diagnosis on the schizophrenia spectrum was selected instead. Additionally, collection of clinical data in the current context also hinges on whether there was an outpatient follow-up appointment that coincided with the corresponding milestone of the patient’s follow-up with EPIP; if the patient did not attend the appointment or an appointment was not scheduled, clinical data for that time point would not be captured. Future work should also look into the possibility and validity of extrapolating continuous data in order to overcome the rigidity of having fixed assessment time points.

Despite the authors’ best efforts, the lack of complete weight and clinical data of a portion of the data sets collected restricts the current study from developing beyond a descriptive paper, which in turn limits the generalizability of its findings and the extent of conclusions that can be drawn from it. Nevertheless, preliminary results still prove illuminating and worthy of discussion, and provide justification for future studies to prospectively capture and monitor metabolic activity according to international consensus standards, as well as pharmacological, dietary, and physical activity data. This will aid in early detection of risk for severe weight gain and understanding of its mechanisms, in order to tailor weight interventions effectively and maximize treatment outcomes beyond symptomatic remission. As reported by Garrido-Torres and colleagues (37), antipsychotic treatment may not be the only factor involved in altered metabolic outcomes; genetic factors and social adversity, or other variables not yet explored, may predispose or exert influence on patients towards obesity. For example, Alameda and colleagues found that patients who experienced psychological trauma during adolescence had a greater waist circumference after 1 year of antipsychotic treatment, compared to those who did not experience any trauma (38).

In conclusion, the present study described five different weight gain trajectory patterns over 2-years present in a sample of patients with a diagnosis of first-episode schizophrenia spectrum disorder, and contributed to the limited knowledge on individual weight profiles from treatment in a specialized early intervention service. Weight interventions are crucial for those at-risk for or already with severe weight gain, but are too costly to be implemented indiscriminately. As these interventions are only truly cost-effective when targeted at those who are at a greater need, identifying this group of high-risk patients as accurately and as early on as possible is imperative to minimize the side effects of antipsychotic medications while maximizing the treatment benefits for these patients (39). In addition, the present study found that existing metabolic monitoring standards were still discrepant from recommended guidelines, and further highlighted the impetus to minimize this gap. Future research directions should include prospectively evaluating challenges in implementing these monitoring practices in detail, such as qualitative interviews or focus group discussions from patients’ and hospital staff’s perspectives, using statistically robust methods (such as latent class group analysis) to elicit discrete weight gain trajectories with complete data sets which include pharmacological, dietary, and physical activity data, and also continuing to explore the mechanisms behind antipsychotic-induced weight gain beyond type of antipsychotic prescribed.

## Data availability statement

All the data from this study reside with the Office of Research, Institute of Mental Health. Data are not available for online access; however, readers who wish to gain access to the data can write to the Clinical Research Committee, Institute of Mental Health/Woodbridge Hospital Secretariat at IMHRESEARCH@imh.com.sg. Access can be granted subject to the Institutional Review Board (IRB) and the research collaborative agreement guidelines. This is a requirement mandated for this research study by our IRB.

## Ethics statement

The studies involving human participants were reviewed and approved by National Healthcare Group Domain Specific Review Board (Ref. No.: 2020/01388). Written informed consent from the participants’ legal guardian/next of kin was not required to participate in this study in accordance with the national legislation and the institutional requirements.

## Author contributions

YC and CT: conceptualization and investigation. YC and EA: methodology, data curation and formal analysis. YC: writing—original draft preparation and project administration. EA and CT: writing—review and editing and supervision. All authors contributed to the article and approved the submitted version.

## Conflict of interest

The authors declare that the research was conducted in the absence of any commercial or financial relationships that could be construed as a potential conflict of interest.

## Publisher’s note

All claims expressed in this article are solely those of the authors and do not necessarily represent those of their affiliated organizations, or those of the publisher, the editors and the reviewers. Any product that may be evaluated in this article, or claim that may be made by its manufacturer, is not guaranteed or endorsed by the publisher.
