# Supplementary material for: A retrospective database study on 2-year weight trajectories in first-episode psychosis
Source: Front Psychiatry. 2023 Jun 27;14:1185874. doi: 10.3389/fpsyt.2023.1185874 (PMC10354663; doi:10.3389/fpsyt.2023.1185874)
Supplement: Supplementary file 1 [file Table_1.DOCX]

Supplementary Table 1. Two-year clinical and functioning scores among the five weight trajectories.

|  | Super high risk | High risk mitigated | At risk | Delayed risk | Low risk | *F* | *p*-value |
| --- | --- | --- | --- | --- | --- | --- | --- |
| PANSS scores at 2-year – mean (SD)   - Total - Positive - Negative - General psychopathology   GAF disability score at 2-year – mean (SD) | 42.3 (12.7)  8.9 (3.3)  11.6 (6.3)  21.8 (6.2)  71.4 (9.4) | 40.9 (11.8)  9.0 (3.2)  10.7 (5.1)  21.2 (5.9)  72.4 (10) | 41.2 (11.7)  8.7 (2.4)  10.8 (5.7)  21.7 (6.4)  74.2 (9.6) | 49.7 (18.3)  11.0 (5.1)  12.7 (7.2)  25.9 (8.1)  66.9 (11.6) | 44.4 (14.9)  9.5 (3.3)  11.3 (5.6)  23.5 (7.6)  70.8 (10.6) | 3.067  0.987  4.323  3.24  2.439 | 0.017^*^  0.414  0.002^**^  0.012^*^  0.047^*^ |
| ^*^*p*<0.05; ^**^*p*<0.01.  PANSS: Positive and Negative Syndrome scale; GAF: Global Assessment of Functioning. | | | | | | | |

Supplementary Table 2. Linear regression with two-year PANSS total score as the dependent variable.

|  | *B* | 95% CI | *p*-value |
| --- | --- | --- | --- |
| Age  Gender  Tertiary highest education level  DUP  Multiple inpatient admissions  PANSS total score at baseline  GAF disability score at baseline  Weight trajectory group   - Super high risk - High risk mitigated - At risk - Delayed risk - Low risk | -0.077  -0.471  1.447  0.016  3.457  0.040  -0.068  -8.068  -9.422  -8.318  Ref.  -5.185 | -0.30 – 0.14  -3.14 – 2.20  -1.44 – 4.33  -0.05 – 0.08  0.74 – 6.18  -0.04 – 0.12  -0.21 – 0.07  -13.05 – -3.09  -14.47 – -4.38  -16.12 – -0.52  -  -10.87 – 0.50 | 0.488  0.729  0.325  0.626  0.013^*^  0.308  0.341  0.002^**^  <0.001^**^  0.037^*^  -  0.074 |
| ^*^*p*<0.05; ^**^*p*<0.01.  DUP: duration of untreated psychosis; PANSS: Positive and Negative Syndrome scale; GAF: Global Assessment of Functioning. | | | |

Supplementary Table 3. Linear regression with two-year GAF disability total score as the dependent variable.

|  | *B* | 95% CI | *p*-value |
| --- | --- | --- | --- |
| Age  Gender  Tertiary highest education level  DUP  Multiple inpatient admissions  PANSS total score at baseline  GAF disability score at baseline  Weight trajectory group   - Super high risk - High risk mitigated - At risk - Delayed risk - Low risk | 0.024  0.297  -0.868  -0.013  -2.435  -0.011  0.055  4.985  5.864  7.076  Ref.  3.869 | -0.14 – 0.19  -1.71 – 2.31  -3.05 – 1.31  -0.06 – 0.03  -4.49 – -0.38  -0.07 – 0.05  -0.05 – 0.16  1.23 – 8.75  2.06 – 9.67  1.19 – 12.96  -  -0.42 – 8.16 | 0.775  0.772  0.434  0.585  0.020^*^  0.717  0.305  0.009^**^  0.003^**^  0.019^*^  -  0.077 |
| ^*^*p*<0.05; ^**^*p*<0.01.  DUP: duration of untreated psychosis; PANSS: Positive and Negative Syndrome scale; GAF: Global Assessment of Functioning. | | | |
